# Empowering indigenous women in Guatemala: a case study of the role of Digital Community Centers in enhancing digital literacy and changing gender perspectives in Northern Huehuetenango

**DOI:** 10.3389/frma.2025.1488916

**Published:** 2025-04-29

**Authors:** Nereyda Y. Ortiz Osejo, Susana Arrechea, Alejandro Alvarado

**Affiliations:** New Sun Road, P.B.C., Richmond, CA, United States

**Keywords:** Mayan women, women's empowerment, digital skills, GEM scale, GNDR-4 scale

## Abstract

**Introduction:**

This study examines how Digital Community Centers (DCCs) contribute to the empowerment of indigenous Mayan women in Northern Huehuetenango, Guatemala. Although rural and indigenous communities remain largely excluded from digitalization, the DCC model aims to narrow the digital gap by providing internet access, basic computer training, and workshops on positive masculinities.

**Methods:**

We employed a mixed-methods approach, including 10 semi-structured focus groups and 43 surveys. The survey assessed digital literacy and gender attitudes using the GNDR-4 and GEM scales.

**Results:**

Findings show significant improvements in women's digital skills after a short training period. These gains enabled participants to reduce travel time for tasks such as processing government documents and to launch small-scale economic initiatives. Participants who attended the positive masculinity training—both men and women—reported more equitable attitudes toward women's leadership and decision-making.

**Discussion:**

Despite these gains, participants stressed ongoing barriers—most notably limited infrastructure, constrained financial resources, and insufficient institutional support—that hamper the long-term viability of the DCCs. They also noted a need for more detailed and standardized training on gender topics to sustain changes in attitudes over time. In conclusion, DCCs offer a promising strategy for bridging the digital divide and facilitating women's socio-economic participation, but further research with larger samples and longer follow-up periods is warranted to confirm and expand upon these initial findings.

## 1 Introduction

Digital literacy and access to technology are powerful catalysts for social and economic development, affecting everything from education to healthcare and beyond (World Bank, 2016; UNESCO, 2015). However, a profound digital divide persists, particularly in rural and marginalized communities, hindering access to essential resources and creating additional barriers for women (Hafkin and Huyer, 2006). These challenges become even more acute in indigenous settings, where historical inequalities and limited infrastructure further restrict women's educational and economic opportunities (Zaremberg, 2024).

Although digital tools have shown their potential to bolster growth and reduce poverty, rural communities often remain on the periphery of such advancements (Van Dijk, 2006; Hilbert, 2011). Poor infrastructure, scarce financial resources, and lower educational attainment contribute to systemic cycles of marginalization. In response, Digital Community Centers (DCCs)—also referred to as telecenters—have emerged as a viable approach to bridging the gap. By situating internet connectivity, computers, and training programs directly within underserved communities, DCCs help address logistical barriers and foster community engagement (Barton and Bear, 1999; Bailey and Ngwenyama, 2013). This can be particularly impactful for women, who face additional cultural and socioeconomic constraints that limit their ability to benefit from digital technologies.

This study focuses on the remote Chuj region of Huehuetenango, Guatemala, to examine how DCCs advance indigenous women's empowerment. Specifically, we ask: how does access to the internet, digital literacy contribute to the empowerment of indigenous women in rural Guatemala? Empowerment, particularly for women in rural contexts, is commonly defined as a process through which individuals gain the ability, resources, and agency to make strategic life choices (Kabeer, 1999). In this study, we conceptualize empowerment both at the individual level—where new digital skills enable women to manage administrative tasks, acquire and share knowledge, and assume leadership roles—and at the collective level, where stronger relationships emerge among female leaders, local authorities, and educational institutions. We address this question by drawing on a mixed-methods approach that captures both the qualitative experiences of indigenous women and the quantitative shifts in empowerment-related indicators. Our contributions are threefold: (1) shedding light on an often-overlooked demographic—Mayan women in remote rural settings; (2) extending the discourse on how digital education intersects with women's agency in contexts shaped by limited resources; and (3) offering a combined qualitative-quantitative analysis that helps illuminate the nuanced impact of DCCs under real-world constraints such as small sample sizes, language barriers, and sporadic community engagement.

The remainder of this paper is structured as follows: we begin with a description of our methods, detailing the selection of participants, data collection procedures, and analytical techniques. We then present the findings, first from the qualitative analysis of focus group discussions, followed by the quantitative assessment of changes in digital literacy and women's empowerment. Finally, we discuss the broader implications of these results for sustainable, community-led development, acknowledge limitations, and propose directions for future research.

## 2 Background

Numerous studies underscore the transformative potential of telecenters, also known as Digital Community Centers (DCCs), in bridging the digital divide and fostering socio-economic development in rural areas. Telecenters not only grant internet connectivity and access to information but also serve as platforms for community capacity-building, thereby encouraging education and economic activities (Díaz Andrade and Urquhart, 2009). In Latin America, early work highlighted how telecenters boosted digital inclusion and advanced social services in underserved regions, offering critical resources and skills to local populations (Proenza et al., 2001).

Within Central America, specific initiatives illustrate the varied ways DCCs can drive rural development. Panama's InfoPlazas and Costa Rica's Intelligent Community programs, for instance, demonstrate measurable improvements in digital literacy and entrepreneurial activities in remote communities (Proenza et al., 2001). Their successes underscore that strong community involvement, combined with partnerships involving local organizations, is vital for sustaining telecenter projects (Cecchini and Scott, 2003). These programs also show that targeting women's participation is particularly critical, as persistent cultural norms and limited mobility can otherwise curtail women's access to technology (Hafkin and Odame, 2002).

Empowering women and girls through digitalization is broadly recognized as an essential strategy for inclusive development. When women gain digital skills, they are better positioned to participate in the workforce, access health information, and advocate for their rights (Heeks and Molla, 2009). Nonetheless, multiple challenges persist, including financial barriers, inadequate infrastructure, and prevalent stereotypes that constrain women's entrepreneurial ventures (Akpuokwe et al., 2024). Further complicating the picture, sociocultural beliefs about gender roles can shape everything from pro-environmental behaviors (Xia and Li, 2023) to political participation and online safety (Setiyaningsih et al., 2023). Taken together, these factors highlight the need for gender-responsive policies and training that address the specific contexts and obstacles rural women face.

Measurement tools like the Gender Equitable Men (GEM) and GNDR-4 scales offer insights into changes in household, economic, and political empowerment—factors that can be directly influenced by DCC-based interventions. Hilbert (2011) found that women's access to information and communication technologies boosts economic and political engagement in rural communities. Likewise, Proenza documented how telecenters in Latin America equipped women with new skills and pathways to local governance, emphasizing telecenters' role in bridging the gender digital divide. Additional research reveals that digital competencies can serve as a powerful mechanism for upward mobility among other marginalized groups as well (Liu et al., 2024; Worcester, 2021), suggesting the broader applicability of DCC-based models that integrate skill-building, community engagement, and ongoing support.

While this study specifically focuses on Northern Huehuetenango, it is important to note that New Sun Road (NSR) has also implemented solar-powered, internet-enabled DCCs elsewhere in Guatemala—particularly in Alta Verapaz and Quiché. Across these broader initiatives, NSR has reached more than 4,000 Q'eqchi', Chuj, and Ixil-speaking women, offering digital skills training and promoting gender-equitable norms. Although these other regions are not included in the current analysis, they provide a valuable context: past successes and lessons learned reinforce the potential for DCCs to empower rural communities in diverse sociolinguistic and geographic settings.

## 3 Data and methods

Our data comes from two sources, interviews and surveys that aim to answer the main research question: how do Digital Community Centers contribute to the increase in digital skills and change in gender perspectives among indigenous women in Northern Huehuetenango? Field staff from New Sun Road, who share the ethnic background and language of participants, conducted the surveys in person, and the interviews online. One of their primary responsibilities was to ensure that participants clearly understood each question. The field staff collected responses verbally and organized them in a spreadsheet. All participants provided informed consent, and steps were taken to ensure their confidentiality and anonymity throughout the study. The role of the field staff was crucial because of the trust they had built through the broader Mujer Prospera project, under which the surveys and digital tests were administered. This trust was vital for addressing sensitive gender-related questions in rural areas where traditional gender norms often prevail.

### 3.1 Qualitative approach

This study employed a qualitative methodology to explore participants' experiences with the Digital Community Centers (DCCs) in Guatemala and to gain deeper insight into shifting gender perspectives and digital inclusion in indigenous communities. Through semi-structured focus group interviews, we aimed to understand the perceived impact of the DCCs, areas for improvement, collaboration among community members, and local priorities related to digital skills and gender norms. These focus groups provided a space where participants could express their views collectively, fostering an environment that encouraged dialogue and reflection on shared experiences. The decision to conduct focus groups rather than individual interviews was based on the understanding that participants generally feel more comfortable speaking in a group setting. However, we remained aware of potential group leader bias, where dominant voices might influence the discussion. To mitigate this, facilitators were trained to ensure that all participants had the opportunity to share their perspectives, using targeted follow-up questions and structured turn-taking strategies.

A total of 10 focus groups were conducted, comprising 33 Mayan women and 10 Mayan men, all of whom were 18 years or older. Participants were selected randomly from 10 different communities in Huehuetenango, ensuring representation across diverse community settings. The sample included community leaders, members of the Women's Leadership Committee (WLC), and other key stakeholders who had benefited from the DCCs and had varying degrees of involvement in leadership and training initiatives. The focus group discussions were conducted virtually via Google Meet, allowing participants to join from their respective DCCs while minimizing travel burdens. To accommodate linguistic diversity, staff from New Sun Road provided live interpretation from Mayan Chuj to Spanish, ensuring accurate communication and comprehension. Facilitators were trained in culturally sensitive interviewing techniques to encourage open dialogue and minimize potential power imbalances that could arise in discussions about gender norms and community leadership. In addition, focus groups with women were led by indigenous female facilitators, while groups with men were led by indigenous male facilitators, ensuring that discussions took place in a setting aligned with participants' cultural and gender-specific comfort levels.

In determining the number of focus groups, we continued recruitment until thematic saturation was reached—that is, until new data no longer yielded additional themes. Recruitment also involved consultation with local community leaders to ensure a diverse range of ages, marital statuses, and leadership backgrounds among participants. All procedures received ethical clearance, and each participant provided informed consent before the session commenced. Where literacy levels were low, the study team read consent forms aloud and answered questions to ensure full understanding.

Each session began with an introduction and an icebreaker activity to establish rapport and ease participants into the discussion. The facilitators then guided the conversation in the native language using a semi-structured interview format, starting with broad, open-ended questions, and progressively narrowing the focus toward more specific topics related to the DCCs' benefits, gender dynamics, and digital literacy challenges. This approach allowed participants to share their perspectives freely while also ensuring consistency across focus groups. The interview guide, which included key questions and themes explored during the discussions, is available in the Supplementary material. Participants were given explicit assurances of confidentiality, and permission for recording was obtained at the beginning of each session. Discussions were audio-recorded and later transcribed for analysis.

A thematic analysis approach was employed to identify recurring patterns in participants' responses. The transcriptions were coded using an inductive-deductive hybrid approach, where inductive coding allowed themes to emerge directly from participants' narratives, while deductive coding was guided by key research questions on gender norms, digital literacy, and community engagement with the DCCs. Two independent researchers reviewed the transcripts and applied coding frameworks to enhance intercoder reliability and ensure the robustness of the analysis. The findings revealed several key themes, including the perceived benefits of the DCCs, such as increased access to technology, improved digital skills, and greater confidence in using digital tools for communication and employment. Challenges and barriers also emerged, particularly regarding connectivity issues, limited local expertise, and persistent gender-based constraints that affected women's ability to participate fully. The discussions also highlighted shifts in gender perspectives, particularly regarding shared responsibilities in digital literacy training and evolving attitudes toward leadership roles for women in the community. Additionally, participants emphasized the importance of community-led solutions and sustainability, noting that long-term success would require local ownership, continued training, and ongoing support from community organizations.

By integrating virtual focus groups, multilingual facilitation, and a structured analytical approach, this qualitative component provided rich, context-specific insights into how digital literacy initiatives intersect with gender equality and digital literacy efforts in indigenous communities. The findings complement the quantitative survey data, offering a nuanced understanding of how digital and social interventions interact in rural Guatemala.

### 3.2 Quantitative approach

For our quantitative analysis, we surveyed 10 men and 33 women who self-identify as Mayan. The field staff administered two surveys—the Gender Equitable Men (GEM) scale and the Gender Equality (GNDR-4) scale—with baseline data collected in October 2022 and endline data in August 2023 under the USAID-funded Mujer Prospera project in Huehuetenango. The same respondents participated in both baseline and endline for these scales. For the digital test, we only included women who took part in GEM and GNDR-4, totaling 29 women at baseline and 32 at endline. The baseline for the digital test was collected in October 2022 and the endline in August 2023, also as part of USAID-Mujer Prospera, administered exclusively to women because they were the project's main beneficiaries and the ones receiving the digital skills training.

The intervention included training on positive masculinities, intended to shift attitudes toward gender roles, and a digital skills training program that spanned roughly 3 months. In these traditional communities, the workshop discussions primarily framed “women” from a biological perspective, reflecting local cultural norms, and practices. The positive masculinities training was based on deconstructing traditional mandates associated with hegemonic masculinity, emphasizing shared responsibility and respect for women's rights. Through participatory activities, it highlighted how certain gender roles perpetuate inequality and how challenging they can foster more equitable, violence-free relationships. The session also underscored the need for everyday practices that support social justice and equality, urging both individual and collective commitment to transforming patriarchal structures.

The digital skills curriculum was designed to improve proficiency in communication, information, problem-solving, and content creation, following Eurostat's Digital Skills Indicator (European Commission, 2015) framework. Participants learned how to use basic Microsoft Office tools, navigate the internet, perform Google searches, handle government documentation online, and even experiment with emerging AI applications, such as ChatGPT and Bing, for daily tasks.

The digital literacy test administered by the field staff was adapted from the Eurostat Digital Skills Indicator (European Commission, 2015) to capture foundational digital competencies in rural communities where routine access to computers and the internet can be limited. To accommodate the linguistic and cultural context, the field staff conducted the test orally in participants' primary languages, ensuring they fully understood each question or task. Participants were asked about specific activities they had performed in the past 3 months across four key domains—information, communication, problem-solving, and content creation.

1. **Information** included tasks such as conducting online searches or identifying reliable digital sources.
2. **Communication** encompassed activities like sending messages via email or chat platforms and participating in video calls.
3. **Problem-solving** covered using digital tools to address everyday challenges—e.g., filling out online forms, locating government services, or troubleshooting basic technical issues.
4. **Content creation** involved creating or editing documents, spreadsheets, or other digital materials that went beyond simple data entry, reflecting a higher skill level.

Each domain was evaluated on a three-tier scale—“none,” “basic,” or “above basic”—depending on whether and how many domain-specific tasks participants reported completing. When participants indicated they had never performed a given task or lacked the resources to do so, they were classified at the “none” level. Those who had done at least one relevant activity were typically classified as having “basic” skills, while those who performed tasks requiring more advanced functions—such as editing documents collaboratively, creating presentations, or handling more complex online processes—were placed at the “above basic” level. These domain-specific scores were combined into an overall digital skills assessment that reflects participants' general competencies. Because the Eurostat methodology emphasizes performance on tasks completed within the last 3 months, it was better suited to documenting the practical digital experiences of our participants.

To assess shifts in gender perspectives, we utilized two validated survey instruments to measure changes in participants' gender-related attitudes and evaluate the impact of an intervention aimed at fostering gender equality and promoting positive masculinities. Please refer to Supplementary material for the full methodology and questions on each survey. The first instrument was the Gender Equitable Men (GEM) Scale, adapted to measure progress toward one the key performance indicators for the Mujer Prospera Project: the percentage of women and men trained who demonstrate attitudes supportive of more equitable gender norms. Originating from a widely validated, cross-cultural scale, the GEM Scale assesses attitudes across multiple domains, including gender roles, sexual behavior, violence, masculinity ideals, and reproductive health. For each domain, statements such as “The most important role of a woman is to take care of her home and cook for her family” or “A woman should tolerate her partner's violence to keep her family together” are presented, and respondents indicate their level of agreement on a Likert-type scale (e.g., *totally agree, somewhat agree*, or *disagree*). Each answer is then assigned a point value, with higher scores signifying stronger support for gender-equitable norms. Summing individual responses yields a composite score, which can then be converted to a 0-to-1 range (0 = extremely low support for gender equality; 1 = extremely high support) for ease of interpretation. This transformation facilitates tracking of overall change and simplifies reporting to funding agencies. In line with the recommended approach from the original GEM methodology, the total number of items was culturally adapted to reflect local priorities and norms without overburdening respondents.

The second instrument used was the GNDR-4 Survey, known as the “Equal Opportunity Survey,” which aligns with other key performance indicators for the Mujer Prospera Project: the percentage of participants who report increased agreement that men and women should have equal access to social, economic, and political resources. The survey presents three key statements, including (1) “Women should have the same rights as men and receive the same treatment,” (2) “In general, men are better political leaders than women and should be elected instead of them,” and (3) “When jobs are scarce, men should have more right to a job than women.” Respondents rate their agreement on a four-point scale (e.g., *totally disagree* to *totally agree*). Each item is coded from −2 (totally disagree) to +2 (totally agree), with statements marked “(r)” scored in reverse so that higher totals consistently denote more egalitarian attitudes. The summed score can then be compared between baseline and endline to evaluate changes in participants' views of gender equality.

Both surveys were administered at two time points (pre- and post-intervention) by trained field staff who shares the language and cultural background of the participants. This was critical for establishing rapport, ensuring respondents understood each item clearly, and maintaining confidentiality—especially important when covering sensitive topics such as gender violence, traditional norms, or personal beliefs about masculinity. For participants with limited literacy, enumerators read questions aloud in their local language, and recorded responses to mitigate misunderstandings. Each respondent's data were entered into a secure database, and personally identifiable information was masked or encrypted to preserve anonymity.

This study employed a before-and-after design to evaluate shifts in both gender-related attitudes and digital competencies, using the same participants at baseline and endline for a paired analysis. A total of 43 respondents (33 women and 10 men) provided complete data for the GNDR-4 and GEM scales, while 32 women participated in the digital skills assessment at both time points. The GNDR-4 and GEM surveys measured various dimensions of gender equality, masculinity, and violence, whereas the digital test evaluated communication, information, problem-solving, and software proficiency.

Although each survey item—and each digital skill category—was ordinal in nature, composite scores were generated for GNDR-4 and GEM by summing item-level responses, facilitating the calculation of average changes across sex, education, marital status, and parental status. For the digital test, individual skill levels also ranged from “none” to “above basic,” enabling a paired comparison of pre- and post-intervention performance. By using these summative or ordinal measures, the analysis could capture overall shifts in attitudes and competencies, rather than focusing on individual item responses alone.

To test for statistically significant changes, the Wilcoxon Signed-Rank Test was applied to both the survey items (GNDR-4 and GEM) and the digital literacy categories. This non-parametric test is well suited to paired ordinal data and does not assume a normal distribution, making it ideal for evaluating pre- and post-intervention responses. The findings are illustrated in figures that display differences in gender attitudes and digital skill levels, offering insight into the extent and direction of change attributable to the intervention.

## 4 Results

### 4.1 The impact of Digital Community Centers through the lenses of Mayan women

This section presents the findings from focus groups conducted in several communities in Northern Huehuetenango, focusing on the impact and challenges of Digital Community Centers (DCCs).

The introduction of Digital Community Centers (DCCs) has significantly improved access to essential services, digital literacy, and economic opportunities across San Mateo Ixtatán and Nentón. The DCCs have provided crucial services such as energy and computer access, photocopying, and processing of government documents like birth certificates and national IDs, which have notably reduced travel time and expenses, particularly benefiting women with young children. One participant shared, “Before the DCC, I had to travel 4 h to get a document for my child. Now, I can do it in 10 min here in the village.”

Beyond these essential services, the DCCs have played an important role in improving digital literacy and skill development, especially among women. This has empowered them to acquire new knowledge, manage local administrative tasks, and contribute more effectively to community development. “Before I did not know how to manage a computer, now we created a Facebook page to promote *El Cenote*....our touristic place.” In some communities, the availability of energy through the DCCs has enabled new economic activities, such as small businesses that rely on electricity, further contributing to community empowerment. “Now, 23 are selling popsicles and renting the fridge for keeping birthday cakes refrigerated.” During the rainy seasons, when home energy solutions are often limited, the DCCs have provided a reliable source of power and connectivity, underscoring their value in these remote areas.

Despite the clear benefits, several challenges were consistently identified across the communities. Many DCCs face significant resource shortages, including inadequate training for female leaders, limited internet connectivity due to the absence of hotspots, women leader's rotations due to migration, traditional gender roles, lack of time, or interest, and insufficient equipment. These limitations restrict the centers' ability to expand their services and meet the growing needs of the communities. For instance, one community member noted, “The people in the community want to have internet in their houses and the hotspot does not reach all the community.”

Logistical issues, such as the need for physical infrastructure improvements—like installing secure doors or enhancing security measures—have also led to temporary closures and reduced the effectiveness of some centers. In two communities, a lack of commitment and communication between community leaders and the Women's Leadership Committee (WLC) was highlighted as a major concern, leading to inconsistent service provision and poor management. Furthermore, the transition of community leaders has, in some cases, resulted in a loss of engagement and support for the DCCs, weakening their impact and sustainability.

Using an inductive–deductive thematic analysis of focus group transcripts, we identified the theme of Collaboration and Community Engagement as key to the DCCs' impact. During coding, participants' own words and narratives were organized under “community relationships,” “local leadership,” and “institutional support,” allowing us to track how DCCs shape and are shaped by communal structures. On one hand, the inductive component enables codes and themes to emerge directly from participants' narratives, without imposing preconceived theoretical frameworks (Braun and Clarke, 2006). On the other hand, the deductive component is guided by predetermined categories and assumptions drawn from existing literature or specific research objectives (Fereday and Muir-Cochrane, 2006). By integrating both approaches, this analysis captures novel insights arising from the data while also assessing consistency with established theoretical constructs, thereby enhancing the study's overall validity and depth.

From these discussions, it emerged that DCCs have generally fostered improved communication and collaboration within communities, strengthening relationships between female leaders, local authorities, and educational institutions. This collaboration has been instrumental in advancing local development efforts. However, this positive impact has not been uniformly experienced across all communities. Some areas have reported limited engagement from local leaders and a lack of clear communication regarding the DCCs' progress and needs. As one leader remarked, “We need more consistent support and communication from our local authorities to keep the DCC running smoothly.”

To enhance collaboration, participants consistently suggested increasing dialogue with schools to better promote the DCCs' services and involve a broader range of stakeholders in the decision-making process. “Now, we (the women's leadership) collaborate with the teachers to provide services to the students, also communicate with the Cocodes (community leaders) to provide support for their meetings; they are asking us to become part of the Cocode,” noted one participant. By integrating direct quotations into our coded categories, we observed that establishing clear communication channels and assigning well-defined responsibilities are seen as crucial steps for sustaining the centers' positive impact and ensuring continued community support.

Although the primary focus of this research is on digital literacy and women's empowerment, participants consistently emphasized that reliable energy and small-scale economic ventures (such as selling cold products) are integral to making Digital Community Centers (DCCs) sustainable. From the community's perspective, access to energy not only supports the digital services offered but also creates local revenue streams that can, in turn, reinforce women's autonomy and decision-making.

To ensure the long-term sustainability of the DCCs, several key recommendations emerged from the focus groups. First, there is a strong desire to expand the range of services offered by the centers. Suggestions include introducing commercial activities, such as selling cold products that take advantage of the energy that the Centers have. Providing educational programs for children in collaboration with local schools was also mentioned. Only the DCCs with enough income for investment have been able to buy a fridge that responds to this request. Not in all the communities teachers and women's leadership committees have agreed to work together.

Enhancing internet connectivity by installing hotspots and upgrading the existing network, alongside providing additional training on how to use the internet for accessing information, and communicating with relatives and productivity, are considered essential steps to improve the quality and range of services. Furthermore, communities have emphasized the need for ongoing training and capacity-building for female leaders to ensure they can effectively manage the DCCs and continue to drive community development.

Finally, consistent engagement and communication between community leaders, the WLC, and local authorities were repeatedly emphasized as critical to the sustainability and growth of the DCCs. Implementing these recommendations will not only address the current challenges but also position the DCCs as central hubs for community development, ensuring they continue to deliver significant benefits to the residents of San Mateo Ixtatán and Nentón.

### 4.2 Women's empowerment and digital skills training and the subsequent changes in these communities

This section presents the quantitative results from the GNDR-4 and GEM surveys conducted in Northern Huehuetenango, as requested by USAID to assess the project's impact, along with the results from digital literacy tests. We analyze both pre- and post-intervention data across all three instruments and explore the association between women's empowerment and digital skills. Although the sample size is limited, requiring further observations, these initial findings offer valuable insights into the project's effects.

To contextualize these results, Table 1 presents a summary of the participants' demographic characteristics. The sample consists primarily of women, with varying levels of education, marital status, and ethnic backgrounds. Given that many participants have limited formal education and come from communities where digital access is historically restricted, their baseline digital literacy levels, and gender attitudes provide critical reference points for understanding the intervention's impact.

**Table 1:** Descriptive summary of sample.

**Demographic variable**	**Male (*n*^a^ = 10)**	**Female (*n*^a^ = 33)**
**Marital status**		
Married	8 (80%)	13 (39%)
Single	2 (20%)	20 (61%)
**Has children**	8 (80%)	13 (39%)
**Education level**		
No education	3 (30%)	9 (27%)
Elementary incomplete	4 (40%)	6 (18%)
Elementary school	3 (30%)	15 (45%)
Middle school	0 (0 %)	1 (3%)
High school	0 (0%)	2 (6.1%)
Ethnicity		
Chuj	10 (100%)	27 (82%)
Mam	0 (0%)	1 (3%)
Mestizo	0 (0%)	5 (15%)

a n (%). Source: Authors' own calculations, 2023.

#### 4.2.1 Digital skills

**Figure 1:**
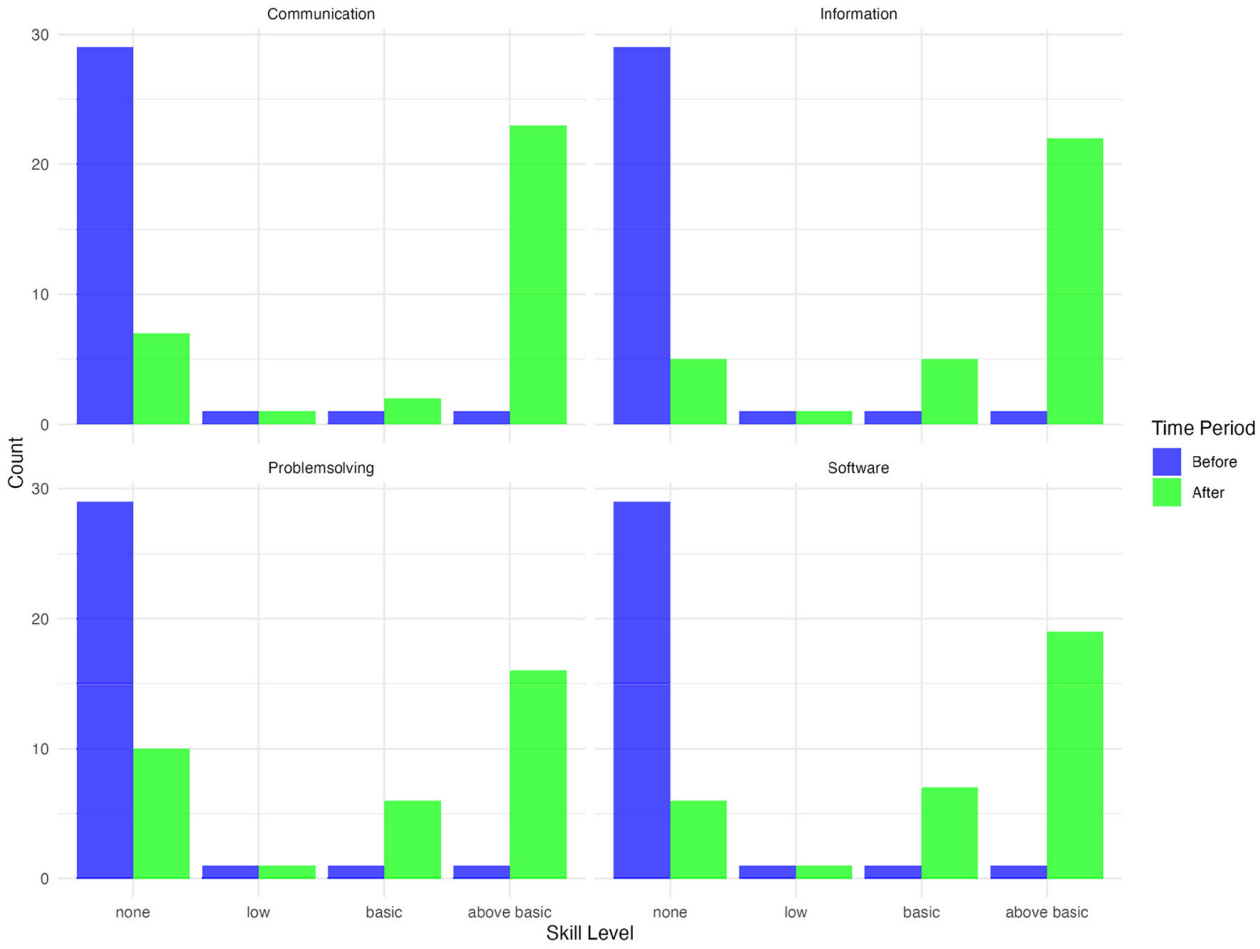
Digital skills by level before and after intervention. Source: Authors' own calculations, 2023.

Figure 1 presents the distribution of digital skills across four domains—Communication, Information, Problem-Solving, and Software—before and after the intervention. The bars represent the number of participants at different skill levels (none, low, basic, and above basic) at both time points, with blue indicating the pre-intervention period and green representing the post-intervention period.

A clear shift toward higher skill levels is observed across all four domains. Before the intervention, the majority of participants had no digital skills in all categories, as shown by the dominance of blue bars in the “none” category. However, after the intervention, there was a notable reduction in the number of participants with no skills and a corresponding increase in those achieving basic and above-basic skill levels.

Specifically for Communication and Information skills, before the intervention, nearly all participants lacked communication and information-related digital skills. After the intervention, a substantial proportion of participants progressed to above-basic levels, indicating an increased ability to use digital tools effectively for communication and information retrieval. For Problem-Solving and Software skills, a similar trend is evident in these domains, where post-intervention results show an increased number of participants attaining basic and above-basic competencies. This suggests that the training significantly improved their ability to navigate digital challenges and use software applications more effectively.

Table 2 presents the results of the Wilcoxon Signed-Rank Tests for digital skills, showing significant improvements across all assessed domains. The analysis reveals statistically significant changes in self-reported competencies before and after the intervention. Given the ordinal nature of the data, the Wilcoxon Signed-Rank Test was used to assess shifts in participants' skill levels across four categories: Information, Communication, Problem-Solving, and Software. The results indicate highly significant improvements in all domains (*p* < 0.001), suggesting that the training was associated with substantial gains in digital literacy.

**Table 2:** Wilcoxon Signed-Rank Test results for digital skills.

**Digital skill**	***n* (paired)**	**Median before**	**Median after**	**Wilcoxon V**	**p-value**	**Effect size (*r*)**
Information	29	1	4	0	4.28e−06^***^	0.881
Communication	29	1	4	0	4.61e−06^***^	0.864
Problem-solving	29	1	3	0	4.52e−05^***^	0.808
Software	29	1	4	0	1.06e−05^***^	0.857

^*^p < 0.05, ^**^p < 0.01, ^***^p < 0.001. Source: Authors' own calculations, 2023.

The median scores for all four skills increased notably—each moving from a pre-intervention median of 1 (none) to a post-intervention median of at least 3 (basic) or 4 (above basic). This upward shift suggests that participants not only acquired basic competencies but also gained sufficient confidence to perform more advanced tasks, such as evaluating online information and navigating diverse software tools. These differences were statistically significant, as evidenced by the small *p-values* and substantial effect sizes (e.g., *r* = 0.81– 0.88). Such high effect sizes underscore the practical importance of these changes, implying that the training meaningfully enhanced participants' digital literacy across all skill domains.

These results indicate that the intervention had a statistically significant impact on enhancing digital competencies across all skill areas. The findings suggest that participants not only gained foundational digital skills but also advanced their abilities to navigate and engage with technology in more complex ways. These results underscore the effectiveness of the training in bridging digital gaps and promoting digital inclusion among the participants.

#### 4.2.2 GEM scale

**Figure 2:**
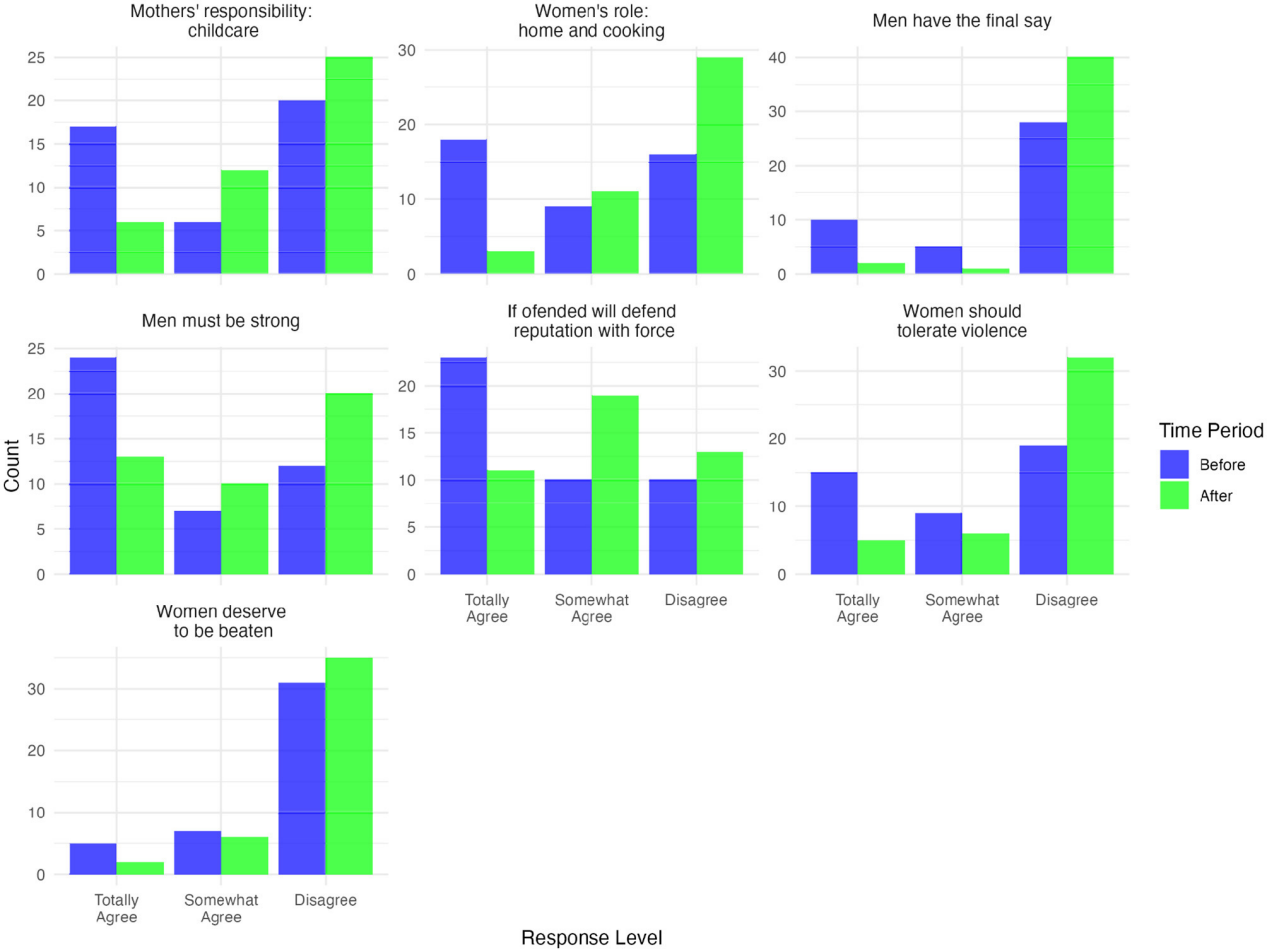
Number of responses for GEM scale before and after intervention. Source: Authors' own calculations, 2023.

The analysis of the GEM scale provides insights into how participants' attitudes toward gender-equitable norms, particularly in areas related to gender roles, violence, and masculinity, changed following the intervention. The training on positive masculinities aimed to encourage reflection on traditional beliefs, and the results presented in Figure 2 indicate notable shifts in responses.

One of the most apparent changes was observed in attitudes toward gender roles. Before the intervention, a substantial proportion of respondents expressed agreement with statements reinforcing traditional expectations, such as “The most important role of a woman is to take care of her home and cook for her family” and “Changing diapers, bathing, and feeding the children is the mother's responsibility.” Following the intervention, there was an increase in the number of respondents who “Totally disagree” with these statements, suggesting a movement away from rigid, gender-based domestic roles. This shift may indicate a greater openness to more flexible understandings of caregiving responsibilities.

Changes were also observed in attitudes toward violence and masculinity. Prior to the training, a considerable number of participants agreed with statements such as “A woman should tolerate her partner's violence to keep her family together” and “To be a man, you have to be strong.” After the intervention, a higher proportion of respondents “Totally disagreed” with these statements, suggesting a shift in perspectives regarding the acceptability of violence and traditional notions of masculinity centered on strength and dominance. While these changes in responses indicate a rejection of certain traditional norms, further research would be needed to assess whether these shifts reflect longer-term attitudinal change.

The broader trends in response distributions, suggest an overall movement toward more progressive attitudes post-intervention. While individual perspectives on gender roles and masculinity are shaped by multiple factors and may not change immediately, the observed shifts in responses provide preliminary evidence that participants engaged with the themes presented in the training and may have reconsidered certain traditional norms. These findings highlight the potential of targeted training programs to facilitate discussions about gender roles and masculinity in contexts where these norms are deeply embedded, for more information see Supplementary Figure A1.

**Figure 3:**
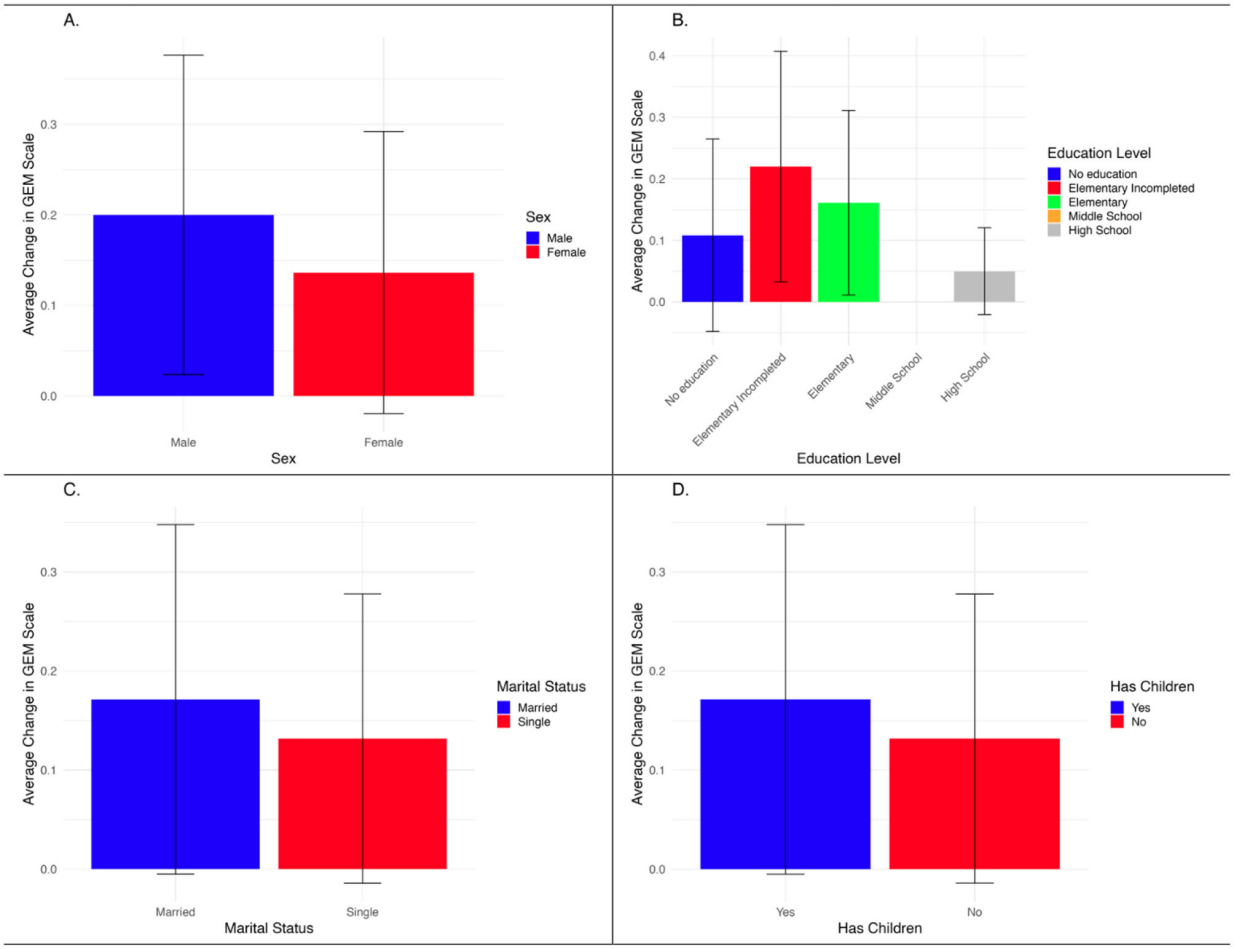
Average change GEM scale by demographic variables. Source: Authors' own calculations, 2023. **(A)** Sex. **(B)** Education level. **(C)** Marital status. **(D)** Has children.

The analysis of the GEM scale, presented in Figure 3, analyzes the average change in attitudes toward gender-equitable norms across demographic groups. Although the confidence intervals indicate that these differences are not statistically significant, they suggest areas for further exploration. Specifically, men exhibited slightly greater shifts in GEM scores compared to women, particularly in attitudes related to masculinity and caregiving. Education level also appeared to influence responses, with those having some or completed elementary education showing the highest positive changes, though the differences were not statistically strong. Notably, the bar for individuals with Middle School education is missing in the figure because no respondent of the GEM survey reported that level of education. Marital and parental status played a minor role, with married individuals and parents demonstrating slightly higher shifts, possibly reflecting their engagement in household dynamics.

Table 3 presents the Wilcoxon p-values for the GEM scale items, offering insights into changes in attitudes toward gender roles, masculinity, and violence following the intervention. The response categories for these items are ordinal, where a score of 1 indicates total agreement with the statement, 2 indicates partial agreement, and 3 indicates disagreement. Given the ordinal nature of the response categories, the Wilcoxon Signed-Rank Test was used to assess whether these changes were statistically significant. This non-parametric test was chosen because it is appropriate for paired data when comparing pre- and post-intervention responses without assuming a normal distribution. The results indicate that several items showed statistically significant shifts, suggesting a potential influence of the training on participants' views.

**Table 3:** Wilcoxon Signed-Rank Test results for GEM items.

**GEM item**	***n* (paired)**	**Median before**	**Median after**	**Wilcoxon V**	**p-value**	**Effect size (*r*)**
Cook and home	43	2	3	51.0	0.00066^***^	0.523
Children's responsibility	43	2	3	108.5	0.04690^*^	0.322
Men have last word	43	3	3	11.0	0.00444^**^	0.503
Tolerate violence	43	2	3	28.5	0.00195^**^	0.502
Women beaten	43	3	3	15.0	0.09730	0.237
Men must be strong	43	1	2	82.0	0.01470^*^	0.375
Reputation	43	1	2	122.0	0.05580	0.326

* p < 0.05,

** p < 0.01,

*** p < 0.001. Source: Authors' own calculations, 2023.

The median values on several items moved higher (e.g., from 2 to 3 or from 1 to 2), signifying decreasing agreement with traditional statements about women's roles, men's decision-making power, and the acceptability of violence. For example, the statement “*Man should have the final say about decisions in his home”* (“Men Have the Last Word”) showed no change in the median score (remaining at 3), yet the Wilcoxon test indicated a statistically significant shift in the distribution of responses (*V* = 11.0, *r* = 0.503). This suggests that while the central tendency did not shift, many participants adjusted their responses in a more egalitarian direction, potentially moving from stronger to more moderate agreement or toward disagreement. The statement “*Most important role for a woman is to take care of her home and cook for her family”* (“Cook & Home”) showed a marked increase in median (from 2 to 3), reflecting more equitable views on domestic responsibilities. The Wilcoxon V values for these items were relatively high, suggesting that the observed changes were not only statistically significant but also broadly shared across participants. Furthermore, the effect sizes ranged from moderate to large (e.g., *r* = 0.38 – 0.52), reinforcing the conclusion that the intervention had a meaningful impact on reshaping these gender-related attitudes.

Although most items showed significant changes, a few did not. The statement “*There are times when a woman deserves to be beaten”* (“Women Beaten”) did not pass the significance threshold, indicating that while some participants shifted their views on this topic, the change was not as pervasive as for other items. Similarly, the statement “*If someone insults me, I will defend my reputation, even by force if necessary”* (“Reputation”) failed to reach statistical significance, suggesting that reducing the importance placed on men's public image may require additional time or more targeted interventions.

The Wilcoxon Signed-Rank Test detects changes in the distribution of responses but does not establish causality. The observed patterns may suggest an association between the intervention and changes in views on masculinity, domestic roles, and the acceptability of violence. However, some items did not show significant movement. These findings underscore the potential of targeted interventions to foster more equitable attitudes, particularly in areas where initial views were more traditional. Future research should explore whether these shifts are sustained over time and whether they correspond with changes in behavior at the household or community level.

#### 4.2.3 GNDR-4 scale

**Figure 4:**
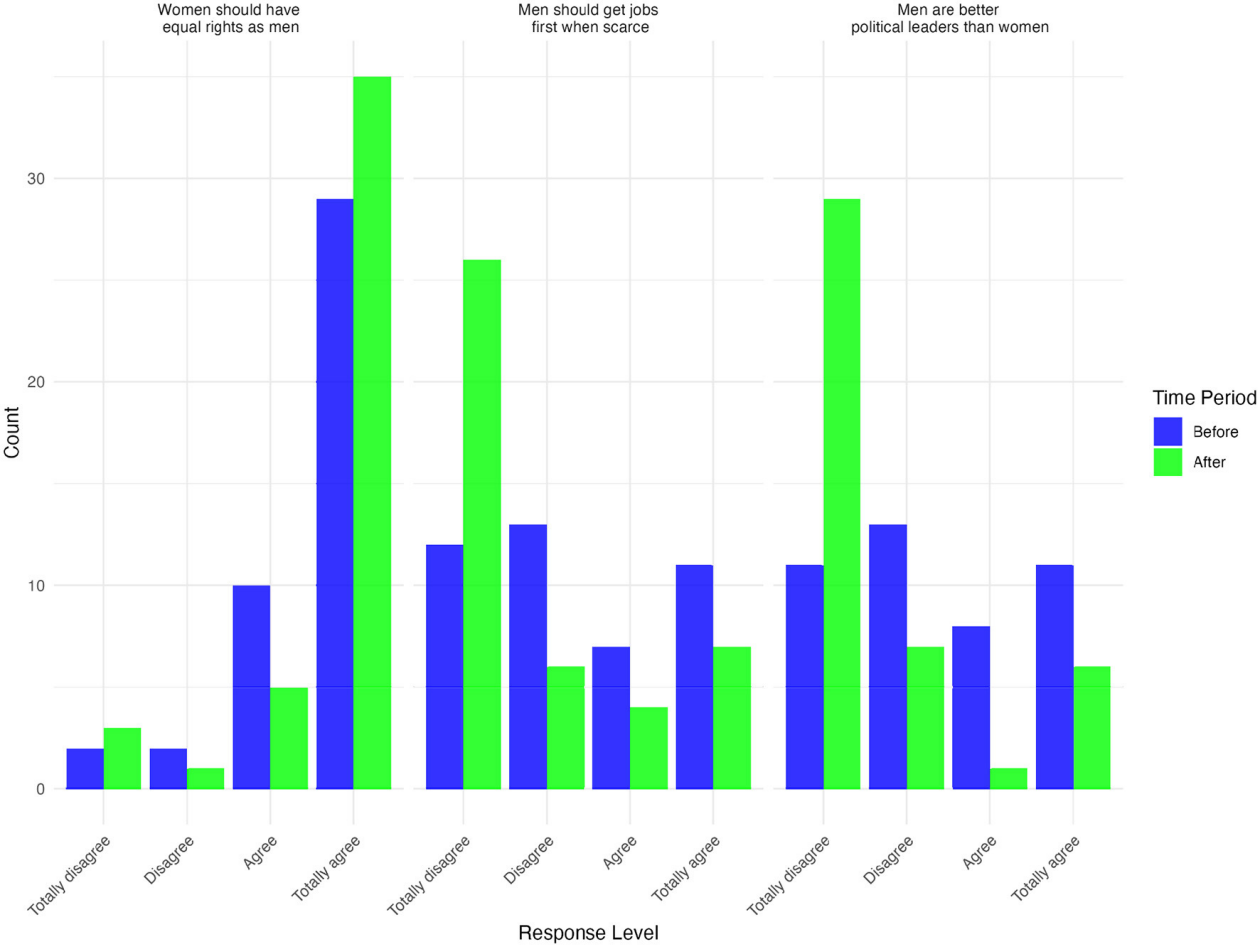
Number of responses for GNDR-4 scale before and after intervention. Source: Authors' own calculations, 2023.

The results from the GNDR-4 scale analysis reveal significant shifts in attitudes toward gender equality following the intervention on positive masculinities. The training aimed to challenge traditional gender norms and promote more equitable perspectives. The findings, illustrated in Figure 4, show meaningful changes in respondents' views regarding women's rights, economic opportunities, and political leadership.

Before the intervention, the majority of respondents either “Agreed” or “Totally agreed” with the statement that women should have equal rights as men. However, post-intervention, there was a noticeable increase in the number of respondents who “Totally agree,” signaling a stronger endorsement of gender equality. A similar trend is observed in responses to the statement “Men should get jobs first when scarce.” While a significant proportion of participants initially supported or were neutral on this notion, post-intervention responses showed a marked increase in the “Totally disagree” category, indicating a rejection of the idea that men should be prioritized over women in employment during times of scarcity. This shift suggests that the training successfully challenged deep-rooted economic biases that favor men in the workforce.

A related change occurred in attitudes toward political leadership. Prior to the intervention, responses to the statement “Men are better political leaders than women” were mixed, with a considerable number of respondents expressing agreement. However, post-intervention, there was a clear increase in those who “Totally disagree,” reflecting a growing rejection of the belief that men are inherently better suited for leadership roles. This shift suggests that the training not only influenced participants' views on economic equality but also challenged traditional notions of political representation and leadership.

These changes in attitudes are further illustrated by the overall distribution of responses on the GNDR-4 scale. As shown in Supplementary Figure A2, the density distribution reveals a notable shift from before to after the intervention. Prior to the intervention, responses were highly concentrated in the lower range of the scale (around 8–10 points), suggesting relatively low endorsement of gender-equitable norms. After the intervention, the distribution flattened and extended further toward higher values, indicating greater variability and a general movement toward more progressive attitudes. This post-intervention shift implies that participants, on average, endorsed more gender-equitable views after exposure to the training. While the earlier distribution sharply peaked, the broader spread in the after group suggests a more widespread uptake of non-traditional gender norms across participants.

Taken together, these findings suggest that the intervention was effective in fostering more egalitarian gender perspectives, particularly in areas related to economic participation and political leadership. While further research is needed to assess the long-term sustainability of these attitude changes, the results highlight the potential of targeted training programs in shifting deeply ingrained societal norms.

**Figure 5:**
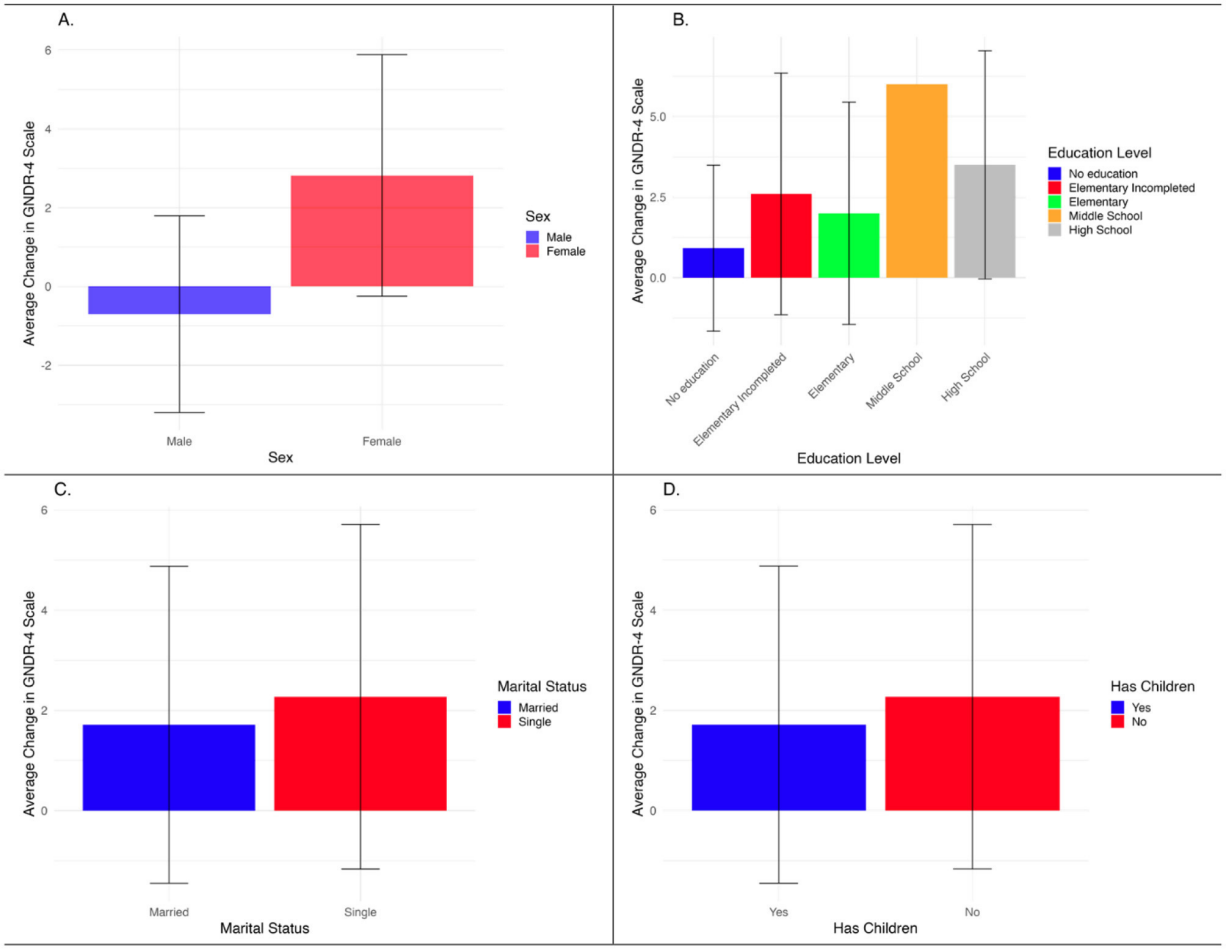
Average change GNDR-4 scale by demographic variable. Source: Authors' own calculations, 2023. **(A)** Sex. **(B)** Education level. **(C)** Marital status. **(D)** Has children.

The analysis of the GNDR-4 scale by demographic variables, shown in Figure 5, displays the average change in attitudes across sex, education level, and marital status. While trends suggest that women, individuals with higher education, and single participants exhibited more progressive views post-intervention, the confidence intervals indicate that these differences are not statistically significant. The lack of statistical significance suggests that the observed variations may be due to sample variability rather than the intervention's direct impact. Notably, the “Middle School” education group includes only one respondent, which prevents calculation of a standard deviation and thus results in the absence of an error bar for that category. These findings highlight the need for further research with a larger and more balanced sample to determine whether the intervention effectively influences gender equality attitudes across different demographic groups.

To assess whether the intervention led to significant changes in attitudes toward gender equality, we conducted Wilcoxon Signed-Rank Tests on the GNDR-4 scale items. This test was chosen because it is suitable for ordinal Likert-scale data and evaluates whether the distribution of responses before and after the intervention significantly differs. Unlike parametric tests, Wilcoxon does not assume a normal distribution, making it an appropriate choice for non-normally distributed ordinal data.

Table 4 presents the results of the Wilcoxon Signed-Rank Test for each of the GNDR-4 items, measuring shifts in attitudes toward gender equality before and after the intervention. The analysis reveals statistically significant changes in two out of the three items, particularly in attitudes toward political leadership and labor rights. The original response scale ranged from −2 to 2, where −2 indicates totally disagree, −1 disagree, 1 agree, and 2 totally agree. However, two items—“*Men are better political leaders than women”* and “*Men should get jobs first when scarce”*—were reverse-coded prior to analysis to ensure consistent interpretation across items. Specifically, responses were recoded so that higher values reflect greater disagreement with gender-inequitable statements (e.g., −2 became 2, −1 became 1, 1 became −1, and 2 became −2).

**Table 4:** Wilcoxon Signed-Rank Test results for GNDR-4 items.

**GNDR-4 item**	***n* (paired)**	**Median before**	**Median after**	**Wilcoxon V**	**p-value**	**Effect size (*r*)**
Equal rights	43	2	2	78.0	0.297000	0.194
Political leadership	43	−1	−2	392.5	0.000867^***^	0.521
Labor rights	43	−1	−2	249.0	0.019200^*^	0.322

* p < 0.05,

** p < 0.01,

*** p < 0.001. Source: Authors' own calculations, 2023.

The most pronounced shift was observed in the item on political leadership (“Men are better political leaders than women”). Responses moved from a median of −1 (agree) to −2 (totally agree) on the reverse-coded scale, indicating increased disagreement with this traditional gender norm. The change was statistically significant (*p* = 0.00087) with a large effect size (*r* = 0.521), suggesting that participants became more supportive of women's leadership following the intervention. Attitudes regarding labor rights (“Men should get jobs first when scarce”) also shifted significantly (*p* = 0.0192), with the median moving from −1 to −2, and a moderate effect size (*r* = 0.322). This suggests a meaningful reduction in agreement with the idea that men should be prioritized in employment during scarcity. In contrast, there was no statistically significant change in the item on equal rights (“Women should have equal rights as men”), with both the pre- and post-intervention median remaining at 2 (totally disagree on the reverse-coded scale). The non-significant *p-value* (*p* = 0.297) and small effect size (*r* = 0.194) suggest that participants already held strong egalitarian views on this issue prior to the intervention, leaving limited room for further improvement.

Since two of the three *p-values* are below the conventional 0.05 threshold, the results suggest that the observed changes in responses are unlikely to be due to random variation alone. The findings indicate that the intervention may be associated with shifts in attitudes toward gender equality, particularly in relation to political leadership and labor rights. While general support for equal rights remained high both before and after the intervention, significant changes in more specific beliefs—such as the idea that men are better political leaders or should be prioritized in employment—point to the potential influence of the training on participants' perspectives. However, these findings should be interpreted with caution, as the data do not allow for definitive causal conclusions. Further research, ideally with larger samples and longer follow-up periods, is needed to assess the durability of these attitudinal changes and their implications for behavior in real-world settings.

## 5 Discussion

The findings from this study highlight significant positive outcomes in the GNDR-4 and GEM scales, and Digital Skills tests following the intervention through the Digital Community Centers. These results are essential in understanding the multidimensional aspects of women's empowerment in rural Guatemala.

The intervention led to substantial improvements in digital competencies, with participants showing marked progress in communication, information, problem-solving, and software skills. These enhancements in digital literacy are significant, as they contribute to increased access to information, economic opportunities, and a sense of autonomy among the participants. This suggests that the digital skills training positively influenced women's empowerment in public and economic spheres.

The GEM scale, which focuses on social norms and attitudes, showed positive and significant changes in gender-equitable norms. The GNDR-4 scale, which measures attitudes toward gender equality in rights, economic positions, and political leadership, also saw significant positive and significant shifts. The intervention effectively challenged traditional gender roles and attitudes toward violence and masculinity, indicating a shift toward more progressive views. These findings align with previous research (Heeks and Molla, 2009), which underscores the transformative potential of digital skills for women's economic participation and advocacy.

A key contribution of this study is its extension of the ongoing discourse on the relationship between education, particularly digital education, and women's empowerment. Despite the positive outcomes previously discussed, the study did not find a statistically significant association between changes in digital skills and the GNDR-4 and GEM scales. This lack of statistical significance may be attributed to the small sample size, which could have reduced the power to detect meaningful relationships. Moreover, the high levels of rurality and lower development in the study area might hinder the translation of digital literacy into measurable changes in women's empowerment (Heeks, 2010). These findings suggest that while digital education holds promise, its impact on women's empowerment requires further investigation with larger sample sizes and more rigorous methodologies, such as randomized controlled trials (RCTs).

Another contribution of this paper is the mixed-methods approach, which is particularly valuable in development projects conducted in challenging contexts (Bamberger et al., 2010). By employing a mixed-methods approach that integrates qualitative insights from focus groups with quantitative data from the GNDR-4, GEM scales, and digital skills assessments, this study provides a more nuanced view of how Digital Community Centers (DCCs) advance women's empowerment. While the quantitative results highlight significant gains in digital skills and shifts toward more equitable gender norms, the qualitative data offer valuable context, revealing that local leadership structures—particularly Women's Leadership Committees—can amplify these benefits by mobilizing resources and strengthening ties with community authorities. Male buy-in to positive masculinities, however, appears to vary by factors such as age and educational background, underscoring the need for tailored approaches to sustain attitudinal changes.

The regression results, provided in the Supplementary Table A1, are not statistically significant, and the direction of the associations between digital education and women's empowerment varies depending on the survey. Increased digital education appeared to positively influence empowerment in terms of rights, economic status, and political leadership (as measured by GNDR-4). Conversely, it seemed to have a negative relationship with gender norms in the private sphere (as measured by GEM). This contrast suggests that while digital education may empower women in public and economic domains, it could also challenge traditional gender norms in private spheres, leading to resistance or slower changes. These findings align with the literature, which indicates that different dimensions of women's empowerment can respond differently to similar interventions (Kabeer, 1999; Qian and Li, 2020).

In addition to illustrating the DCCs' direct benefits—such as reducing travel time and enhancing digital literacy—this mixed-methods design underscores the broader, structural elements crucial to making these transformations durable. Although DCCs clearly improve women's daily tasks, broader policy support remains essential to solidify and expand these gains. Infrastructure upgrades, mentorship programs, and ongoing financial backing all emerge as critical pathways for turning the centers into lasting community anchors. By combining robust statistical evidence with in-depth qualitative narratives, the mixed-methods approach captures both the measurable outcomes of digital interventions and the lived realities that influence how—and for whom—empowerment takes shape.

Another major contribution of this study is its focus on Mayan women in rural and remote areas of Northern Huehuetenango, Guatemala—a population that has been largely understudied. This focus provides valuable insights into the specific challenges and opportunities for digital literacy and empowerment within this unique cultural and geographical context. To gain a deeper understanding of these dynamics, future research should explore the impact of digital literacy on women's empowerment with larger and more diverse samples. Conducting RCTs could yield more definitive evidence on the associations between digital education and various aspects of women's empowerment. Additionally, understanding the contextual factors that influence these outcomes, such as cultural resistance and socioeconomic conditions, will be critical for designing more effective interventions (Hafkin and Odame, 2002).

In conclusion, Digital Community Centers and digital training play a critical role in bridging the digital divide and promoting gender equality in rural Guatemala. By providing access to digital technologies and training, DCCs empower indigenous women, fostering economic, and social development. While the study underscores the positive impacts of DCCs, it also highlights the need for continuous and inclusive efforts to address gender disparities. Future interventions should build on these findings, ensuring that digital inclusion strategies are comprehensive, culturally sensitive, and sustainable. Women reported increased confidence in digital literacy and leadership skills, which translated into greater participation in decision-making processes within their communities. The focus groups emphasized the importance of community collaboration and support, as both men and women recognized the value of gender equality and shared leadership roles. Participants also noted the reduction in gender-based barriers and a shift toward more equitable social norms, fostering a more inclusive and supportive community environment.

## 6 Challenges and limitations

While this study provides valuable insights into the impact of Digital Community Centers (DCCs) on indigenous women in Northern Huehuetenango, the small sample size, particularly in the quantitative analysis, limits the generalizability of our findings. The remote nature of the study area and logistical challenges constrained our ability to gather a larger dataset. Future research should consider employing larger sample sizes or randomized controlled trials to validate these initial findings and further explore the relationship between digital literacy and gender empowerment in similar contexts. Expanding the study to include other regions or communities would also help to establish the broader applicability of the results.

Our analysis revealed that the association between changes in digital literacy and gender empowerment, as measured by the GEM scale, was not statistically significant. This lack of statistical significance suggests that the relationship between these variables may be more complex than initially hypothesized. Several factors, such as cultural resistance to changing gender norms or the limitations of the survey instruments in capturing subtle shifts in empowerment, could have influenced these results. Future studies should explore these non-significant findings in greater depth, potentially incorporating additional variables or alternative methodological approaches to better understand the dynamics at play.

Policymakers should prioritize digital inclusion as a cornerstone of rural development agendas, recognizing the multifaceted role that Digital Community Centers can play in empowering marginalized populations. To ensure the sustainability and effectiveness of these initiatives, it is crucial to allocate sufficient resources for infrastructure, training, and ongoing community engagement. Moreover, digital literacy programs should be designed with cultural sensitivity and tailored to address the specific needs of indigenous communities, incorporating feedback mechanisms to adapt to evolving challenges.

## Data Availability

The original contributions presented in the study are included in the article/Supplementary material, further inquiries can be directed to the corresponding author.
