# Supplementary material for: Empowering indigenous women in Guatemala: a case study of the role of Digital Community Centers in enhancing digital literacy and changing gender perspectives in Northern Huehuetenango
Source: Front Res Metr Anal. 2025 Apr 29;10:1488916. doi: 10.3389/frma.2025.1488916 (PMC12069257; doi:10.3389/frma.2025.1488916)
Supplement: Supplementary file 1 [file Data_Sheet_1.pdf]

Supplementary Material

1 Supplementary Data

Questions for focus groups[1]

The specific questions were the following:

A. Community Benefits: Has the Digital Community Center (DCC) benefited your community? If yes, how has it benefited? Can you provide specific examples of how the DCC has been useful to you or your community?

B.Challenges and Areas for Improvement: What do you consider has not worked well with the DCC, and how do you think it could be improved? Are there any services or features of the DCC that you think need more attention or enhancement?

C. Collaboration and Community Engagement: Has the DCC helped foster collaboration between different groups in the community? If so, how? How have you seen community leaders and members working together through the DCC?

D. Sustainability and Future Recommendations: What suggestions or recommendations do you have to strengthen the DCC program and ensure its long-term sustainability? Are there additional services you believe the DCC should offer to better serve your community?

E.Community Needs and Priorities: Are there other priorities or urgent needs that the community considers more important at this moment? How do you see the DCC fitting into these community priorities?

F. Feedback and Additional Comments: Is there anything else you would like to share about your experience with the DCC? Do you have any additional thoughts or recommendations for improving the DCC program?

GEM Survey[2]

Tool for Measuring KPI 2.2

Tool: Gender-Equitable Men (GEM) Scale

KPI 2.2: Percentage of trained men demonstrating positive attitudes toward gender norms.

[1] Focus group design by the authors
[2] Tool provided by USAID-MujerProspera staff

KPI 2.2 Definition: This indicator will be used to evaluate changes in attitudes and gender norms among men participating in a U.S. government-funded norm change intervention. The Gender-Equitable Men (GEM) Scale is a validated and widely used instrument for measuring attitudes towards gender-equitable norms.

Important Information about KPI 2.2:

- The unit of measurement will be a percentage expressed as a whole number.
- Numerator = the number of respondents with strong support for gender-equitable norms.
- Denominator = the total number of respondents.
- Indicator values can range from 0 to 100.
- Data for this indicator will be collected through a survey administered before and after the relevant U.S. government-funded training/program.

About the GEM Scale: The results suggest that the GEM scale is a sensitive and cross-culturally relevant tool with good predictive validity. Adaptations in multiple contexts have worked well.

Number of Subscales: 5

Names of the subscales (or domains): Gender; Sexuality; Violence; Masculinities; Reproductive Health.

IMPORTANT: The following list includes all the statements found in the original GEM scale. You do not need to use them all. We wanted to present the full scale so you could be familiar with it. More information about selecting statements can be found in the "How to Implement" section below.

Items by Subscale

***The items marked with ()* were selected for the GEM Scale in New Sun Road Guatemala.**

Gender

- The most important role for a woman is to take care of her home and cook for her family.*
- Changing diapers, bathing, and feeding children are the mother's responsibility.*
- A man should have the final say about decisions in his home.*

Violence

- A woman should tolerate violence from her partner to keep her family together.*
- There are times when a woman deserves to be beaten.*

Sexuality

- Men need sex more than women do.
- Sex is something you do, not talk about!
- Men are always ready to have sex.
- I would never have a gay friend.
- It is important for a man to have a friend with whom he can talk about his problems.

Masculinities

- To be a man, you need to be tough.*
- Men should feel ashamed if they cannot get an erection.*
- If someone insults me, I will defend my reputation, even by force if necessary.*

Reproductive Health

- It is a woman's responsibility to avoid getting pregnant.
- I would be outraged if my wife asked me to use a condom.
- Both men and women can suggest using a condom.
- If a man gets a woman pregnant, the child is the responsibility of both.
- A man should know what his partner likes during sex.
- The father's involvement is important in raising children.
- A man and a woman should decide together whether they want to have children.
- A man and a woman should decide together which contraceptives to use.

How to Implement:

1. Review the 21 statements included in the original GEM scale methodology, listed above and organized by subscale. Consider which statements should be used for cultural specificity and whether some should be removed if they do not reflect the main gender issues in the target area of your intervention. The number of country-specific statements typically ranges between 11 and 15 (avoid having too many statements).
2. Create a paper or electronic survey with the following introduction: "Please read the statements carefully and indicate to what extent you agree or disagree with each one. There are no right or wrong answers; please respond according to your perception of each statement. I want to assure you again that your responses will not be shared with anyone living in this community, and you do not have to answer any questions you do not want to."

For each statement, include the following predefined responses in your questionnaire: 1) Strongly agree; 2) Somewhat agree; 3) Disagree.
a. Example: Would you say you strongly agree, somewhat agree, or disagree with the following statement? - To be a man, you need to be tough.

i. Strongly agree.

ii. Somewhat agree.

iii. Disagree.

Scoring:

1. To analyze the data, assign 3 points for each response that represents gender equality, 2 points for each response that represents moderate gender equality, and 1 point for each response that represents the lowest level of equality. For example, in the case of the statement "There are times when a woman deserves to be beaten," the response "strongly agree" represents the lowest level of equality and should receive 1 point. The response "disagree" represents the highest level of gender equality and should receive 3 points.

2. For each respondent, calculate the total number of points. The higher the score, the better.
3. The GEM scale should be presented on a scale between 0 (which represents extremely low support for gender equality) and 1 (which represents high support for gender equality). Compared to using higher numbers, this simple scale may be easier to understand for many people.
4. To calculate the scale, divide the respondent's number of points (see step 2 above) by the maximum possible number of points. For example, if 12 questions are used, the maximum score is 36 points (12 x 3 = 36). If a respondent's score of, for example, 29 points is divided by the maximum of 36 points, the GEM scale score will be 0.8.
5. To calculate and report this indicator, the percentage of trained men demonstrating positive attitudes toward gender norms, do the following after scoring:
   a. Calculate the GEM Scale results based on the percentage of men classified as high, medium, or low in terms of overall acceptance of more or less equitable norms.
   b. Indicate the % of respondents who strongly support gender-equitable norms, i.e., the % of respondents who scored in the top third. For example, if you have 12 questions, the maximum score can be 36 points (3 x 12). The "top third" will include respondents who scored between 25 and 36 points, the middle will include respondents who scored between 13 and 24 points, and the bottom third will include respondents who scored between 0 and 12 points.
6. In addition to reporting to USAID, you may be interested in knowing the average score and/or responses to individual questions.
   a. To calculate the average GEM scale score, sum the points obtained by each respondent and divide them by the total number of respondents (excluding those who did not answer one or more questions).
   b. The GEM scale results can also focus on responses to individual questions. For example, "% of men who agree that there are times when a woman deserves to be beaten."

GNDR-4 Survey[3]

Tool for Measuring KPI GNDR-4

Tool: Opportunity Equality Survey

KPI GNDR-4: Percentage of participants who report increased agreement with the concept that men and women should have equal access to social, economic, and political resources and opportunities.

GNDR-4 Definition: This indicator will be used to measure the effectiveness of U.S. government efforts to promote gender equality by assessing changes in attitudes regarding whether men and women should have equal access to resources and opportunities in social, political, and economic spheres. Changes in attitudes are measured through the Opportunity Equality Survey (see Data Source below for survey instructions), administered alongside training or programs in any sector that include goals or objectives related to gender equality and women's empowerment. Projects that aim to change participants' overall attitudes toward gender equality are especially relevant.

Important Information about GNDR-4:

[3] Tool provided by USAID-MujerProspera staff

- The unit of measurement will be a percentage expressed as a whole number.
- Numerator = number of participants whose survey scores have improved over time.
- Denominator = total number of participants who have participated in the relevant training/program.
- Indicator values can range from 0 to 100.
- Data for this indicator will be collected through a survey administered before and after the relevant U.S. government-funded training/program, with one survey at the start and another at the end.

Survey Administration:

- Surveys should be administered to individuals who can be clearly identified as program participants and should be translated into the language(s) spoken by the participants if necessary.
- The survey can be read aloud to program beneficiaries who are illiterate.
- This survey can be combined with another survey you plan to implement at both the baseline (starting point) and the endline (end point) to measure other dimensions of gender equality.

Survey Data Privacy:

- Survey data can be quite personal and may include details about respondents' backgrounds (address, age, marital status, etc.) as well as their thoughts and feelings about certain people and situations. To protect personally identifiable information (PII), PII must be masked, coded, or removed from the dataset and from any shared or published datasets. Ensure that only employees who absolutely need PII to perform their work have access to it, use encryption to keep PII secure, and securely destroy records when no longer needed.

How to Measure:

Create a paper or electronic survey with the following question and three statements. Be sure to explain to respondents, either verbally or in writing, that "there are no right or wrong answers; please respond based on your feelings about each statement. I want to assure you again that your responses will not be shared with anyone living in this community, and you do not have to answer any question you do not want to answer."

Question: To what extent do you agree or disagree with the following statements?

Statements:

1. Women should have the same rights as men and receive the same treatment as them.
2. In general, men are better political leaders than women and should be elected instead of them. (r)
3. When jobs are scarce, men should have more right to a job than women. (r)

Scale:

- Strongly disagree
- Disagree
- Agree

- Strongly agree

Scoring: To evaluate opportunity measures, responses are coded as follows:

- -2 = Strongly disagree
- -1 = Disagree
- +1 = Agree
- +2 = Strongly agree

Items with an (r) should be scored in reverse, meaning items followed by an "r" that have a score of -1 should be recoded as +1. For example, for item 2 ("In general, men are better political leaders than women and should be elected instead of them" (r)), a response of "strongly agree" is recoded as "-2".

A higher score indicates greater agreement that men and women should have equal opportunities.

Reporting Timeline:

GNDR-4 results should be reported at the end of the training/program, when changes in attitudes can be calculated.

Questions for the Digital Literacy test[4]

Digital Skills Indicator – Derived from the Eurostat Survey on ICT Usage by Individuals

Methodological Note - 2015

Recognizing the crucial role of digital competence in today's society, the European Commission's Digital Agenda for Europe 2010 dedicated an entire pillar to digital literacy, skills, and inclusion. Additionally, recognizing the need for indicators to measure the degree of digital competence in Europe, one of the actions of the Digital Agenda was to "propose by 2013 EU-wide indicators of digital competence and media literacy" (action 62).

Following a report from May 2014, DG CONNECT and the Eurostat Information Society Working Group agreed to create and publish a "Digital Skills Indicator" based on the Digital Competence Framework (developed by the JRC and DG EAC, available for self-assessment on the Europass website), which would be populated with data collected through the ICT survey on household and individual ICT usage.

The framework identifies five domains of competence: information, communication, content creation, safety, and problem-solving. The ICT survey collects information on activities conducted in the last 3 months by internet and computer users, covering four of the five domains (the safety domain is not covered as adequate indicators for this domain are not yet available in the survey). It is assumed that people who have performed certain activities possess the corresponding skills.

[4] Eurostat Survey on ICT Usage by Individuals, https://eufordigital.eu/wp-content/uploads/2019/10/Digital-Skills-Indicator-survey-on-ICT-usage-by-Individuals_Methodological.pdf

The nature of the ICT survey does not allow for the investigation of competence levels for each activity performed. However, for each of the four domains, a set of activities (between 4 and 7) has been selected to reflect the competencies outlined within each domain of the Digital Competence Framework, with the purpose of distinguishing between people who have or lack basic skills. When evidence on the variety of tasks performed or their complexity is available, an "above basic" flag is also attributed. Once these three skill levels ("none," "basic," and "above basic") are calculated for each of the four dimensions, an overall composite indicator is calculated following a similar logical approach.

This methodology was first applied using the results of the 2015 survey, and Eurostat will replicate it in future surveys whenever all the necessary variables are available. The Digital Skills Indicator was piloted using a similar approach based on the results of the 2012 and 2014 surveys, using available indicators based on other similar questions. As such, the results of the 2012 and 2014 pilots cannot be adequately compared with the final 2015 indicator results. The overall indicator figures are used by the Commission as part of the Digital Economy and Society Index (DESI) and are presented in the Digital Agenda Scoreboard visualization tool.

Below is the list of the basic "activities performed" indicators used to calculate the digital skills indicators and the criteria used to assign a basic/above basic level:

1. Information Skills

- Copied or moved files or folders
- Saved files in internet storage space
- Retrieved information from public authorities/services websites
- Searched for information on goods or services
- Searched for health-related information

Information Skill Levels:

- Basic: One element
- Above Basic: More than one element

Definition in the Digital Competence Framework: Identifying, locating, retrieving, storing, organizing, and analyzing digital information while assessing its relevance and purpose.

2. Communication Skills

- Sending/receiving emails

Supplementary Material

- Participating in social networks
- Making phone calls/video calls over the internet
- Uploading self-generated content to any content-sharing website

Communication Skill Levels:

- Basic: One element
- Above Basic: More than one element

Definition in the Digital Competence Framework: Communicating in digital environments, sharing resources through online tools, engaging with others and collaborating via digital tools, interacting and participating in communities and networks, and intercultural awareness.

3. Problem-Solving Skills

A - Problem-Solving:

- Transferring files between computers or other devices
- Installing software and applications
- Changing settings of any software, including operating systems or security programs

B - Familiarity with Online Services:

- Online shopping (in the last 12 months)
- Online selling
- Using online learning resources
- Internet banking

Problem-Solving Skill Levels:

- Basic: One or more elements only from A or only from B

- Above Basic: At least one element from both A and B

Definition in the Digital Competence Framework: Identifying digital needs and resources, making informed decisions on the most appropriate digital tools for a given purpose or need, solving conceptual problems through digital means, using technologies creatively, resolving technical issues, and updating one’s own and others' competencies.

4. Software Skills for Content Manipulation

A - Basic:

- Using word processing software
- Using spreadsheet software
- Using software to edit photos, videos, or audio files

B - Above Basic:

- Creating presentations or documents that integrate text, images, tables, or graphs
- Using advanced spreadsheet functions to organize and analyze data (sorting, filtering, using formulas, creating charts)
- Programming in a programming language

Content Creation Skill Levels:

- Basic: None of the "above basic" elements from B
- Above Basic: At least one "above basic" element from B

Definition in the Digital Competence Framework: Creating and editing new content (from word processing to images and videos); integrating and reworking previous knowledge and content; producing creative expressions, media outputs, and programming; handling and applying intellectual property rights and licenses.

Overall Digital Skills Assessment:

- Individuals with an "Above Basic" level of skills:
  - "Above Basic" in all 4 domains
- Individuals with a "Basic" level of skills:
  - At least "Basic" in all 4 domains
- Individuals with a "Low" level of skills (lack of some basic skills):
  - One or more "None" in one to three domains
- Individuals without skills:
  - Four "None" (no items marked in all four domains, despite declaring having used the internet at least once in the last 3 months),
  - And those who used the internet more than 3 months ago or who never used it (*)

(*) The scales can only be calculated for individuals who have used the internet in the last 3 months. Other individuals are outside the scope of the assessment, but when presenting the scales as a percentage of the total population, they can be added to the group of those classified as without skills.

Although the digital skills scale has been updated in Europe to reflect changes and advancements in access to and use of technology, in our context and for our target population in Guatemala—people from rural areas who have not had regular access to a computer—we consider the 2015 scale to be the most appropriate. This version provides an assessment more aligned with the current level of exposure and use of technology in these communities, allowing us to more accurately measure basic digital skills and progress toward digital inclusion.

On Wilcoxon Signed-Rank Test

To enhance transparency and support readers in interpreting the results, we provide additional details on the Wilcoxon signed-rank test used in this study. The test was applied to paired, ordinal data to evaluate changes in participants' responses before and after the intervention. For each item, the test calculates the differences between paired observations, ranks the absolute values of these differences, and then sums the ranks of the positive and negative differences separately. The smaller of these two rank sums is reported as the test statistic (V). We also report the corresponding sample size (n), the median scores before and after the intervention to illustrate the direction of change, and the effect size (r), calculated as $r = \frac{Z}{\sqrt{n}}$, where Z is the standard approximation of the test statistic. This effect size provides a measure of the magnitude of change, complementing the p-value in assessing the substantive significance of the findings.

2 Supplementary Figures and Tables

Figures

Figure A1. Response Distribution for GEM Scale Before and After Intervention

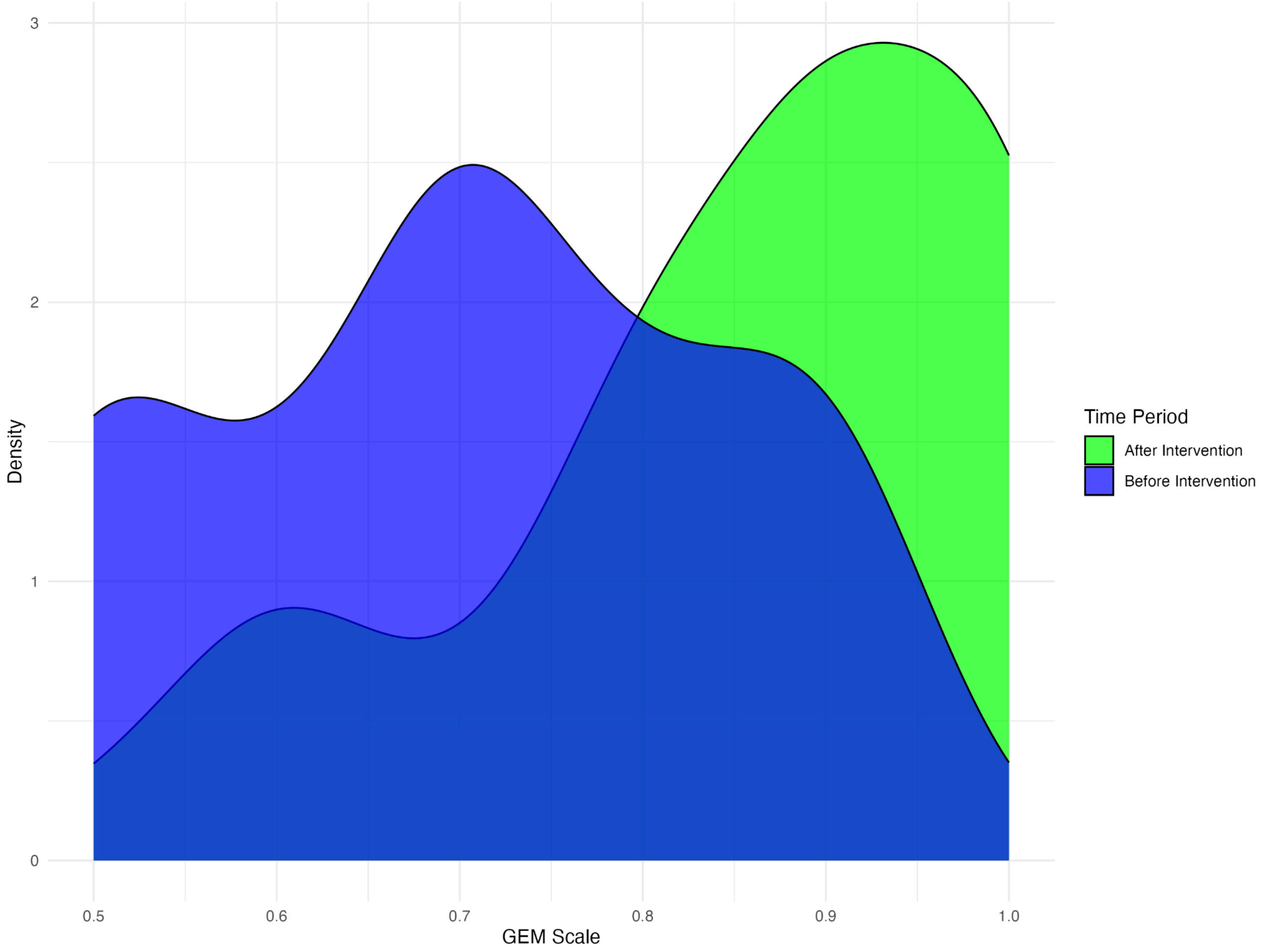

Source: Authors' own calculations, 2023

Figure A2. Response Distribution for GNDR-4 Scale Before and After Intervention

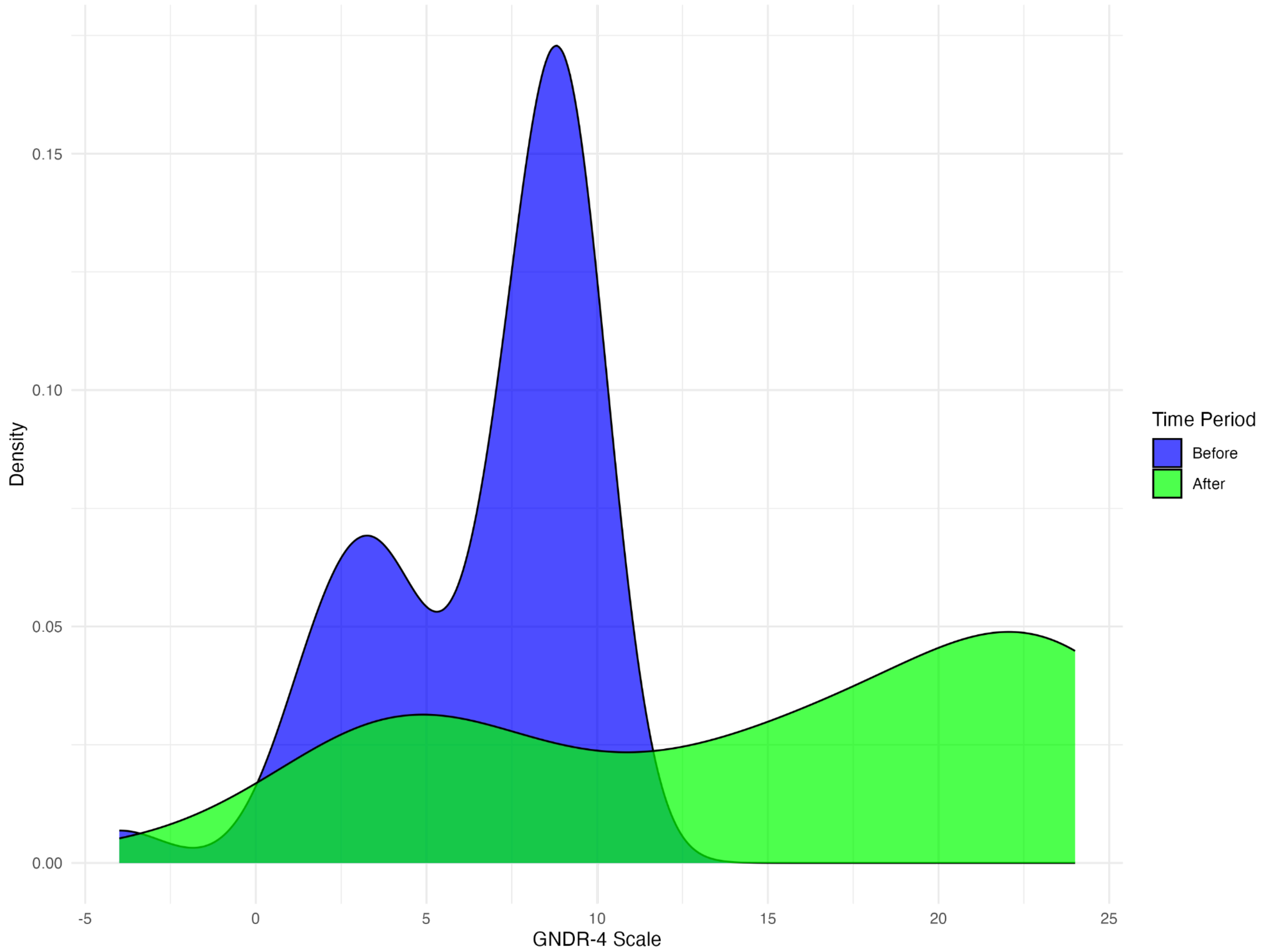

Source: Authors' own calculations, 2023

Tables

Table A1. Association between Women's Empowerment Scales and Digital Education among Mayan Women in Northern Huehuetenango, accounting for demographic characteristics

Variables	Change in GNDR-4 Scale	Change in GEM Scale
Change in General Digital Skills	0.359<br>(4.879)	-0.017<br>(0.031)
Age	-0.056<br>(0.077)	-0.006<br>(0.004)
Education	0.368<br>(0.584)	-0.017<br>(0.031)

Marital	-0.638<br>(1.349)	-0.047<br>(0.072)
Children present	-0.586<br>(1.641)	0.004<br>(0.092)
Intercept	4.350<br>(4.879)	0.420<br>(0.260)
R-squared	0.098	0.108
Adjusted R-squared	-0.075	-0.086
No. of observations	32	32

Source: 2023 GNDR-4 and GEM surveys conducted in Northern Huehuetenango
